# Notes and News

**Published:** 1901-10-05

**Authors:** 


### NOTES AND NEWS.

Sir William Church will open the new pathological laboratory at Oxford on October 12, and many well-known men of science are likely to attend.

Lerwick, in Shetland, has recently acquired a cottage hospital for 12 patients through the generosity of the late Miss Bain and her sister, Mrs. Anderson. The institution is to serve as a memorial to Mr. Gilbert Bain, and to be called after him. Through Miss Bain's bequest and Mrs. Anderson's gift the hospital has been erected and furnished and endowed with a sum of £2,500.

Some of the newly-wakened zeal for the study of tropical diseases is likely to be turned more especially in the direction of beri-beri. The Christmas Islands Phosphate Company have offered £1,000 to the London School of Tropical Medicine if they cause an investigation to be made in the Islands, and Sir John Murray will also support the scheme, and it is hoped that the assistance of the Colonial Office will be obtained.

Although we have heard so much less about it, for more than a year the number of smallpox cases in Paris have exceeded that of the present epidemic in London. If the spread of the disease does not continue, it may do ultimately more good than harm, as it requires some stimulus to urge the public to seek revaccination, and so large a number of people are being re-vaccinated that if the epidemic were to break out next year it will find the population far better able to meet it.

On October 13th Professor Virchow, of Berlin, will celebrate his eightieth birthday. Many festive functions are being arranged in honour of the event. Among other honours to the veteran pathologist the municipality of Berlin has resolved that the great new hospital now being erected in the See-strasse is to bear his name. The Virchow-Krankenhaus will be by far the largest and best arranged of all the municipal hospitals of Berlin. It will contain 1,700 beds for patients, while the medical staff, nurses, servants, and the members of the nurse training institute, which will be attached to it, will bring the number of inmates up to 2,000.

Cases of plague are reported amongst the dock labourers at Punto Franco, Naples.

A cottage hospital has been erected by the employees of the Ebbw Vale Steel, Iron, and Coal Company, assisted by many generous friends. The hospital, which contains ten beds and one cot, cost £3,000. Each member of the medical staff will treat his own patients.

Two new pavilions have been opened at the Ladywell Sanatorium, Salford. They are for the use of convalescents. The larger pavilion will be devoted to those recovering from scarlet fever, to the number of 48, and the smaller pavilion will be used for 32 enteric cases. The buildings are well built and pleasantly decorated.

The peculiar disease called sleeping sickness has claimed so many victims in the islands of Angola and St. Thomé, that a little time ago the Portuguese Government sent a medical commission to study it. The investigations have been successful in discovering the origin of the disease in bacteria, which it is hoped admits of extermination.

The outbreak of smallpox has brought cremation into notice. The Cremation Society of England has made a very excellent offer to cremate persons who have died of smallpox at a merely nominal cost. In no case more than smallpox can cremation be advantageously employed. The highly contagious nature of the disease renders it advisable to destroy effectually all possible means of contamination.

Hospital Saturday, on October 12th, is near at hand. The public must not allow any diminution this year, as the working man is doing his part, as shown by the increased workshop contributions, which are £737 larger this year than last. People in London are no longer annoyed through it by vexatious street begging, and since the administration so wisely abolished this system the fund has advanced in popularity and increased its income.

We learn that the London School of Tropical Medicine have accepted the offer made by Sir Francis Lovell, Kt., C.M.G., Surgeon-General of Trinidad, to undertake a mission to tropical and other countries on behalf of the School, and he will probably leave England in October. Since the school was established on the initiative of Mr. Joseph Chamberlain, M.P., the work done and the number of students attending has far exceeded anticipation and it is now obvious that the facilities afforded to medical men to obtain a knowledge of tropical diseases before taking up their duties abroad has been appreciated not only by the medical profession, but by merchants, planters, and others to whom the health of persons residing in the tropics is of the utmost importance. The school needs now to be placed on a sound financial basis, either by endowment or otherwise, and for this purpose it is hoped that a sum of £100,000 may be raised. Lord Brassey will preside at a meeting at the Royal United Service Institution, Whitehall, on Wednesday, October 16th, at 4 p.m., as a send-off to Sir Francis Lovell, and to deliver an inaugural address on the opening of the third winter session of the school.

